# I Don’t Believe a Person Has to Die When Trying to Get High: Overdose Prevention and Response Strategies in Rural Illinois

**DOI:** 10.3390/ijerph20021648

**Published:** 2023-01-16

**Authors:** Suzan M. Walters, Marisa Felsher, David Frank, Jessica Jaiswal, Tarlise Townsend, Brandon Muncan, Alex S. Bennett, Samuel R. Friedman, Wiley Jenkins, Mai T. Pho, Scott Fletcher, Danielle C. Ompad

**Affiliations:** 1 Department of Epidemiology, School of Global Public Health, New York University, New York, NY 10003, USA; 2 Center for Drug Use and HIV/HCV Research, New York, NY 10003, USA; 3 College of Population Health, Thomas Jefferson University, Philadelphia, PA 19107, USA; 4 Department of Social and Behavioral Sciences, School of Global Public Health, New York University, New York, NY 10003, USA; 5 Department of Health Science, University of Alabama, Tuscaloosa, AL 35487, USA; 6 Department of Population Health, New York University Grossman School of Medicine, New York, NY 10016, USA; 7 Renaissance School of Medicine, Stony Brook University, Stony Brook, NY 11794, USA; 8 Department of Population Science and Policy, SIU School of Medicine, Springfield, IL 62702, USA; 9 Department of Medicine, University of Chicago, Chicago, IL 60637, USA; 10 The Community Action Place, Murphysboro, IL 62966, USA

**Keywords:** overdose, fentanyl, people who inject drugs, harm reduction, polydrug use

## Abstract

Background: Overdose is a leading cause of morbidity and mortality among people who inject drugs. Illicitly manufactured fentanyl is now a major driver of opioid overdose deaths. Methods: Semi-structured interviews were conducted with 23 participants (19 persons who inject drugs and 4 service providers) from rural southern Illinois. Data were analyzed using constant comparison and theoretical sampling methods. Results: Participants were concerned about the growing presence of fentanyl in both opioids and stimulants, and many disclosed overdose experiences. Strategies participants reported using to lower overdose risk included purchasing drugs from trusted sellers and modifying drug use practices by partially injecting and/or changing the route of transmission. Approximately half of persons who inject drugs sampled had heard of fentanyl test strips, however fentanyl test strip use was low. To reverse overdoses, participants reported using cold water baths. Use of naloxone to reverse overdose was low. Barriers to naloxone access and use included fear of arrest and opioid withdrawal. Conclusions: People who inject drugs understood fentanyl to be a potential contaminant in their drug supply and actively engaged in harm reduction techniques to try to prevent overdose. Interventions to increase harm reduction education and information about and access to fentanyl test strips and naloxone would be beneficial.

## 1. Introduction

Overdose deaths have been steadily increasing in the United States (U.S.) since 2013 [1]. In November 2021, the CDC released a communication which used provisional data to report an increase in overdoses of 28.5% from the past year, stating that over 100,000 people died from overdose from April 2020 through April 2021 [2].

Illicitly manufactured fentanyl is now the primary driver of opioid overdose deaths in the U.S. [3]. Fentanyl is a synthetic opioid approximately 50 times more potent than heroin with a rapid onset of action and relatively short duration of effect [4]. Persons who inject drugs may become exposed to fentanyl either intentionally (e.g., specifically seeking its use) or unintentionally (e.g., through undisclosed adulteration of their primary drug) [5,6]. Furthermore, fentanyl has been found in both opioids and stimulants, making opioid overdose prevention and harm reduction important for people who use either or both drugs [7].

In the absence of a safe, regulated drug supply coupled with the criminalization of drug use, people who inject drugs have used a variety of strategies to try to prevent overdose or respond to an overdose in real time. Strategies to avoid overdose include relying on trusting relationships with drug sellers to confirm that drugs are not adulterated with fentanyl [8] and partial drug injection. Partial drug injection comprises several practices, such as “slow shots”, where a person injects a portion of the solution in the syringe, keeping the needle in the injection site, and continuing or withdrawing the syringe, and “tester shots”, where the solution is divided into separate injections [9]. Fentanyl test strips can be used to detect fentanyl as another prevention technique [10]. Test strips enable people to check their drug supply for the presence of fentanyl before consumption by dissolving a small amount of the substance or a sample of drug residue in water [11]. Research has found fentanyl test strip programs to increase awareness about fentanyl and risk reduction behaviors [12,13].

Overdose response techniques include the use of naloxone, an opioid antagonist, to reverse the effects of opioids. From 1996 to June 2014, 136 opioid overdose education and naloxone distribution programs in the US provided naloxone kits to 152,283 people and reported 26,463 potential opioid overdose reversals [14]. These have expanded considerably since then, with a 2019 survey finding that 94% of syringe service programs (i.e., harm reduction service organizations (HRSO)) had implemented an opioid overdose education and naloxone distribution program [15]. However, considerable naloxone deserts exist, especially in rural areas and in states other than California or New York [16]. Even where naloxone is readily available, there are considerable barriers limiting uptake, such as stigma and fear of police arrest, diminishing the potential value of this lifesaving evidence-based intervention [17]. Along with naloxone, research has also identified overdose response techniques such as injecting a person with water or with a salt solution [18,19] and immersing people in cold water [20].

While all states have reported an increase in opioid morbidity and mortality during the past decade, death and injury from opioid use have been concentrated in states with large rural populations [21,22]. Overdose risk in rural locations is further exacerbated by healthcare shortages, underfunding of social services, and longer distances to emergency services [23,24]. There are disproportionately scarce services in rural areas relative to urban areas. For example, there are less opioid use disorder treatment services and providers [25,26,27,28,29], syringe services programs [30], and health department resources for opioid use and mental health [31]. This may further limit rural persons who inject drugs’ awareness of, and access to, overdose prevention resources, particularly biomedical tools such as fentanyl test strips and naloxone.

### Rural Southern Illinois

This study sampled people who injected drugs residing in the 16 southernmost counties of Illinois, referred to as southern Illinois throughout this paper. There is high prevalence of poverty; 45.4% of the residents are living below 200% of the poverty level [32]. Residents are also more geographically isolated compared to other areas of Illinois, with 56.7 residents per mile in southern Illinois compared to 231.1 per mile statewide. The unemployment rate is 9.6% [32,33], and all counties are geographic and/or low income primary and mental health professional shortage areas [34,35]. Furthermore, most health departments provide limited opioid use disorder related services [26].

Past research has characterized this area as at high risk for overdose deaths [36]. In Illinois, a statewide naloxone standing order, as well as bystander protections enacted through the Drug Overdose Prevention Program Law (IL Public Act 096-0361), the Emergency Medical Services Access Law (IL Public Act 097-0678) and the Heroin Crisis Act (IL Public Act 099-0480), serve to facilitate access to naloxone [37]; however, implementation and distribution of the medication remains limited in southern Illinois [26,38].

The increase in fentanyl combined with barriers to healthcare access may leave people who inject drugs in rural Illinois and providers serving them (e.g., drug treatment providers, HRSO, etc.) less prepared to prevent fentanyl overdose [39,40] compared to those in urban locations. This study sought to understand overdose risk and experiences by exploring the context of drug use, perceptions of fentanyl in the drug supply, and overdose prevention strategies in rural southern Illinois. Understanding these contextual factors is critical to address the overdose epidemic.

## 2. Methods

### 2.1. Sample

People who inject drugs were recruited from a local HRSO in southern Illinois and referred in collaboration with research staff conducting a study in the southern Illinois site of the Rural Opioid Initiative (ROI) cooperative agreement (see https://ruralopioidinitiative.org, accessed on 15 November 2022). The HRSO functioned mainly as mobile units that delivered services to people across the 16 most southern counties of Illinois. Eligibility to participate in the study included (a) being 18 years or older, (b) self-reported injection drug use within the last 12 months, and (c) residing in the southernmost 16 counties of Illinois. Eligible people who had agreed to be approached for future research were contacted by study staff and/or HRSO staff and were offered the opportunity to participate in this study. We conducted additional interviews with service providers to provide valuable complementary insight into the community context [41]. The original eligibility criteria for service providers were being 18 years or older and working at agencies who provide services to people who use drugs. Service providers were identified by study staff with local community connections and by the HRSO. These additions were made toward the end of data collection and our sampling was adjusted due to the constant comparative methods we used [42]. Some service providers may have had a history of drug use; however, many of them did not disclose drug use to us during the interview and we did not ask about their drug use.

### 2.2. Research Ethics

All protocols were approved by the institutional review board at New York University.

### 2.3. Data Collection

Nineteen semi-structured interviews were conducted with people who injected drugs and four with service providers from August 2019 through February 2020. Data collection ended due to the COVID-19 pandemic. Therefore, data for this analysis do not contain information about COVID-19. Interviewers were formally trained in ethnographic methods of observation, semi-structured qualitative interviews, writing field notes and memos, and coding. Interviews were audio recorded and ranged in length from thirty minutes to two hours. Written informed consent was obtained from all participants at the beginning of the study visit, and each participant received $40 for their time.

Interviews were conducted in the HRSO’s mobile units or in locations agreed upon by the interviewer and participant. After each interview, study staff immediately created a memo describing their observations and experiences. Memos included details of the interview by the domains explored, body language and tone of the interviewee, observations of the interview site, perceived challenges with interview questions, and any other relevant information that would not come through from a transcript [43,44].

### 2.4. Interview Structure

We used a semi-structured interview guide with open-ended questions that allowed participants to discuss which themes were most resonant for each domain. This strategy also allowed interviewers to probe for additional information depending on participant responses. Interviews focused on the following domains: geographic context of drug markets, perceptions towards fentanyl in the drug markets, and overdose prevention and response techniques used by participants. Separate interview guides were developed for people who injected drugs and service providers, respectively, as described below.

Participants who injected drugs were first asked to contextualize their drug use by answering questions such as, “*Tell me about the types of drugs you use*” and “*How do you get drugs*?” To identify how participants perceived fentanyl in their drug supply, participants were asked, “*Tell me about your experiences with fentanyl.*” Next, interview guides explored the overdose prevention and response strategies employed by participants. To explore overdose prevention strategies, participants were asked to describe their experience with using fentanyl test strips, including the frequency of their use. When participants were unaware of fentanyl test strips, the interviewer provided information about the strips and referred participants to the partner HRSO. The interviewer worked closely with the HRSO to obtain test strips for participants.

To explore experiences with responding to overdoses, we asked, “*Have you ever seen a person overdose*?” If they answered yes, participants were asked to describe the story of what happened when they witnessed an overdose, including how they responded to the overdose. Participants were asked if they used naloxone during this event. When participants witnessed more than one overdose, interviewers encouraged them to tell a detailed story about whichever experience was most salient to them, which was often the most recent overdose experience. We then asked questions about participants’ general experiences with naloxone by asking questions about access to naloxone, comfort carrying naloxone, experiences being trained to administer naloxone, and first-hand experience administering naloxone. Demographic characteristics including age, gender, sexual orientation, education, and employment were collected at the end of each interview.

Service provider guides covered the same domains but asked specific questions relevant to the positions and the organizations that they worked in (e.g., pharmacist, EMS, HRSO staff). Service providers were first asked to contextualize the geographic context of drug use by answering questions such as, “*Tell me about the [city/town],*” *“Tell me about [organization/company] you work at,”* and “*Tell me about how you work with people who inject drugs.*” Service providers were then asked to *“Describe the types of drugs used in [city/town]”* and *“tell me about any overdoses in [city/town].”* They were also directly asked about fentanyl—*“Please tell me what you know, if anything, about fentanyl”*—and fentanyl test strips—*“Can you tell me what you know, if anything, about fentanyl test strips?”*.

### 2.5. Data Analysis

Audio recordings from the interviews were reviewed and professionally transcribed. Informed by grounded theory, constant comparison, and theoretical sampling methods [42], we immediately transcribed the interviews after conducting them and reviewed each transcript to note any immediate themes that arose which might need to be addressed by changing our interview guide or recruitment methods. At that point, the transcript was uploaded to Dedoose (Version 8.3.17) for qualitative coding and assigned to one of two coders [SW and BM]. We coded line by line and then grouped codes into themes. Relevant themes were compiled in a codebook, and the codebook continued to change throughout the coding process as new themes emerged [45]. Codes were reviewed through dialogue about the data and codebook weekly, and a final consensus was reached among the three coders. Once coding was finished, we identified themes related to overdose risk, prevention, and response. Pseudonyms were given to participants to protect their identities when presenting quotes in this manuscript [46].

## 3. Results

We interviewed 19 persons who inject drugs and 4 service providers from rural Illinois. Service provider occupations were as follows: one HRSO staff, two paramedics, and one pharmacist. Demographic characteristics of persons who inject drugs are found in Table 1. Three overall themes emerged from the data: (1) the geographic context of drug use; (2) overdose prevention practices; and (3) overdose reversal practices.

### 3.1. The Context of Drug Use in Rural Illinois—Fentanyl Presence and Awareness

Participants in this study described the drug market as being dominated by methamphetamine. Brandon, a staff member of the HRSO said, “*We recognize...that in southern Illinois we have a lot of meth [methamphetamine] users, and those using methamphetamine...they are injecting, most of them are*.”

Participants were aware that fentanyl had proliferated within their local drug supply—both methamphetamines and opioids—and expressed fear and concern over its effects. For example, Justin, a 58-year-old white man who injected drugs said,

*[Fentanyl is] strong... It’s real dangerous, and...it’s been an epidemic here lately. I tell everybody I know that they need to watch out if they’re trying to purchase something because there’s a lot of fentanyl in the stuff nowadays, and it’s so strong that it’ll kill you if you don’t know what you’re doing.”*

Participants also specifically referenced fentanyl in stimulants. For example, Tawna, a 28-year-old woman said, *“even the ice [methamphetamine] around here could be cut with fentanyl. It could. So, you never know.”*

Participants’ narratives suggest that fentanyl is becoming more common, including in non-opioid substances. Uncertainty about the purity of their drugs caused distress and people took action to try and prevent fatal overdoses.

### 3.2. Overdose Prevention Strategies

#### 3.2.1. Purchasing Drugs from a Trusted Seller: The Importance of Social Networks and Community

Participants perceived that their risk of overdose was significantly lower if they purchased drugs from trusted sellers. Participants described purchasing drugs from trusted drug sellers and having a positive relationship with the person they bought drugs from. One way that drug sellers were perceived to care about the health of drug purchasers was through the secondary distribution of sterile syringes, often distributed with drugs sold. For example, Paul, a 36-year-old white man who injected drugs, explained how he informally supplies new injecting equipment to sellers who then distribute injecting equipment to their clients when they purchase drugs:

*“I got six or seven major dealers in town. They will come to me when I get my shipment, you know, my package of, you know, syringes and stuff, and they will fight to get them. So, they can pass them out with their shit [drugs]. So, we’ve kind of formed a little community.”*

Another participant, Nick, a 44-year-old white man who injected drugs, reported exclusively purchasing drugs from a single seller, or from only a small number of sellers, from whom he or friends had previously purchased from. Nick, said “*I’ve got a decent plug [drug seller] so most of the time, it’s not really an issue you know, it’s good quality.”*

Participants discussed knowing their sellers personally. For example, Alicia, a 44-year-old white woman, said *“I personally know who the dealer is, so I just get it myself. Make a phone call and get it myself.”* Melissa, a 36-year-old white woman, described calling or texting her drug seller and how they would often deliver to her. She then explained how she sometimes used drugs in the presence of her seller, suggesting that sellers and people who use drugs alike can be effective first responders to overdose. Melissa said,

*“If they pick me up, I go back to their house with them and then I end up walking back to my house a couple hours later.”*

Overall participants described having positive personal relationships with people they purchased drugs from. Participants described ways that these relationships could mitigate overdose and infectious disease risks. These included access to new injecting equipment, trust that drugs were uncontaminated and good quality, and, in some cases, a person whom they could use drugs with. Using with another person reduces the risk for fatal overdoses since, if someone overdoses, EMS can be called and/or naloxone can be used.

#### 3.2.2. Modifying Drug Use Practices

Participants also reported adjusting their drug use practices by using small amounts or changing the route of administration if fentanyl was suspected. For example, after having witnessed and rescued many people from accidental overdoses, Justin wanted to minimize his risk of fatal overdose. In response, he consistently used a partial injection strategy and reported no personal history of overdose. He said, “*I make it up, and do a very small amount.”*

Another participant, Steve, a 30-year-old white man who injected drugs, discussed snorting rather than injecting if he was concerned fentanyl was in his heroin. Despite the caution, and the fact that snorting is a harm reduction technique [47,48], overdose can still occur [49]:

*“I bought it earlier in the day and I did a little bit of it and had to be Narcan[ed] so I should’ve known that it was fentanyl, but I didn’t shoot it. I snorted like a little bit of it.”*

Similarly, Alex, a 26-year-old white man told us, *“I keep my shot to a little one”.*

#### 3.2.3. Fentanyl Test Strips

About half of the participants had heard about fentanyl test strips and just under a quarter reported using test strips to detect fentanyl in drug samples. Those that obtained fentanyl test strips likely did so from the HRSO who had been educating about fentanyl and providing test strips in partnership with this study.

Once aware of fentanyl test strips, the majority of participants acknowledged the importance of being able to test their drug samples and expressed interest in using test strips in the future. Jessica, a 30-year-old white woman who recognized that fentanyl was added to many drugs, had heard of test strips but had not used them. Nevertheless, she was interested in starting to use test strips in the future if offered by the study HRSO. Jessica said:

*“You can, like, test the drug that you are doing because now everything’s being laced with it [fentanyl], so, I don’t know much about them”.*

When the interviewer asked Jessica if she plans on using test strips in the future, she replied, “*Yeah, if like though [HRSO]…I would definitely use them every time... I never thought of that. I never that they [the HRSO] did that kind of program*.”

Among the small percentage of participants who used fentanyl tests strips prior to the interview, most had positive experiences with the strips. Nick, who obtained test strips from the HRSO, responded in the following way when asked how often he used the strips:

*“Only when I have a question about it [the drug]. You know, I’ve got a decent plug [drug seller], so most of the time, you know, it’s not really an issue, you know, it’s, it’s good quality. But if there’s ever a question or you know, if it’s ever just not well, you’re not great, you know, I’ll test it. I don’t have any more of them [the strips] now though”.*

David, a paramedic, also explained that fear of being judged negatively or prosecuted are major deterrents to obtaining fentanyl test strips in small-town rural locations:

*“I would say drug users in general, but the needle users especially, are very skittish where they are not going to walk into Walgreens and ask for a fentanyl test kit. They are worried about their phone being GPS’d and the DEA watching them. Because as soon as you do that, then you are going to be on some list and people are going to watch you, people are going to stigmatize… Especially in a small town. Everybody knows everybody. It’s amazing the network that people have... How one gets [test strips] into such a secretive, skittish community would be difficult”.*

### 3.3. Overdose Reversal Practices 

#### 3.3.1. Cold Water and Body Movement

The most common overdose response described by participants in this study was shocking the individual with cold water. For Samantha, a 36-year-old white woman, cold water baths were the only overdose response she was aware of:

*“I’ve seen my daughter’s dad hit the floor, go blue and like, freaked me out. Me and another friend, all we could think of was that put him in the bathtub, turn on the cold water… I’ve been told before, like, tell someone that had overdosed, put him in the bathtub, cold water. You know, ice, whatever get him cold... That’s the only thing I’ve ever been told about an overdose. Put him in the bathtub, turn on pure cold water”.*

Nick described that during his most recent overdose, his friend used a combination of cold water and rescue breathing until he was revived:

*“[I woke] up on the floor covered in fucking dog hair and ice cold, wet towels… Seems like, you know, um, she breathed for me for two hours and maybe like hour and 50 minutes or something. Um, the heart never stopped, but only because she kept good oxygen”.*

Similarly, Fiona, a 44-year-old white woman, describes her husband’s overdose:

*“It was just God-awful. His heart never fully stopped, but his breathing, he was, he was gone. But, um, I shocked him with some ice towels”.*

In addition to cold water, participants tried to keep people moving so they would continue breathing. For example, Justin, a 58-year-old white man who injected drugs, explained his techniques. He said,

*“I don’t believe a person has to die when they’re trying to get high or for whatever reason they are using, it shouldn’t have to kill them. So, I just go into action. I automatically try to get them up and breathing and moving and I’ve been successful every time pretty much…I’ve not had to call the paramedics for anybody.”*

#### 3.3.2. Naloxone

Participants’ descriptions of how they responded to overdoses also revealed barriers to naloxone use, such as fear of the opioid withdrawal. Naloxone was available from the HRSO and could also be obtained at pharmacies without an individualized prescription [50]. Yet, many participants were unaware of naloxone availability, as Samantha explained above when she said that she had only heard of putting people in cold water as an overdose response.

Sometimes participants knew about naloxone, but simply forgot to use it. Bianca, a 44-year-old white woman, described using cold compresses when responding to an overdose and having cold compresses used on her when she overdosed. After discussing overdose experiences, we asked Bianca about naloxone and she replied, *“I forgot about that.”* Bianca then described how she had been trained to use naloxone by the HRSO and someone else had used naloxone on her in the past.

Participants also avoided naloxone because it could cause withdrawal. However, one participant, Steve, a 30-year-old white man who injected drugs, described using naloxone on someone who overdosed and explained why withdrawals did not deter him from using it. Steve received naloxone and was trained to use it by a syringe service provider located in St. Louis, so not the HRSO we partnered with. Steve said:

*“Well I Narcaned him and they got really really mad at me, uh, they came back to life so… I’d rather them be mad at me and alive than dead and not mad.”*

Steve also told us that he had naloxone used on him by EMS or the police (he was not aware which one) the last time he overdosed.

## 4. Discussion

Participants described an awareness that fentanyl had been mixed with drugs within their local drug market. They reported that fentanyl was in both the opioid and stimulant supply, and that they were often unaware of what contaminants might be in the drugs they purchased. At the same time, participants seemed to know very little about overdose prevention and response reduction strategies emerging from many more urban harm reduction efforts, such as use of naloxone and fentanyl test strips. This finding contributes to the growing body of research demonstrating U.S. trends of increased fentanyl in stimulants such as methamphetamines and cocaine [49,51], suggesting the need for overdose interventions tailored to address both opioids and stimulants, as well as polydrug use, in settings that are more rural and may lack harm reduction infrastructure [52].

A first line of defense for avoiding overdoses among participants in this study was purchasing drugs from trusted and consistent drug sellers whom they believed could alert them if fentanyl was in the drugs they purchased. This finding echoes past research highlighting how trusted relationships with drug sellers can lesson overdose risk. For example, Carroll et al., (2020) indicate the majority of participants in their study “spoke about their primary dealers going out of their way to alert clients to the presence of fentanyl or even to avoid selling fentanyl-contaminated product completely [8]”. Bardwell et al., (2019) report similar findings, noting that many participants did not prioritize drug checking if they purchased from a trusted seller [53]. In fact, studies sampling drug sellers have found that they are indeed concerned about the well-being of the people they sell drugs to [54].

Unlike some hierarchal drug markets where the user/buyer often has only infrequent contact with the seller and generally only to purchase drugs, or drug markets dominated by freelance entrepreneurs who often lack interpersonal ties with others in the community, anonymity is more difficulty in many rural settings where face-to-face relationships are often the norm. These drug markets may include socially bonded community relations between people who use drugs and drug sellers, which can be protective; network members—sellers and people who use drugs—are often familiar with each other and others in their extended networks. This can help stabilize product, exerting an informal regulatory control of sorts on drug sales, holding people accountable to others, and ensuring that most people are aware of high- or low-quality drugs [55]. These network relationships can also facilitate the spread of other potentially lifesaving information about overdose prevention and drug checking.

Given the important role that drug sellers play, and the ways in which they can mitigate harms, including drug sellers in overdose prevention and response efforts could be beneficial. Interventions could include drug checking [54] as well as providing drug sellers with fentanyl test strips, new injecting equipment, and naloxone. Additionally, policies that criminalize drug sellers should be reconsidered. Research suggests that laws criminalizing drug sellers do not reduce access to drugs. Imprisoning trusted drug sellers could push people to buy drugs from unknown sellers, which could increase overdose risk [56].

Participants also described using smaller amounts of drugs and/or changing the route of administration to smoking or snorting to prevent overdose. Past research has also found using smaller amounts, sometimes called “test shots” [57,58], and changing the route of administration [59] to be effective harm reduction techniques.

We also asked participants about fentanyl test strip knowledge and use. About half of the sample had heard of test strips and just under a quarter had used them. A study sampling people who use drugs in 2018 living in rural West Virginia found just over 10% of participants had heard of fentanyl test strips [57]. Our study sample may have more awareness because of the HRSO since most participants had heard of strips and/or obtained them from the HRSO. Thus, another possible intervention could be mail-order naloxone and fentanyl test strips so people living in spaces without HRSO access might be able to obtain the harm reduction tools they need [60].

This study also points to the important role HRSOs and syringe service programs play in the lives of people who use drugs, and how they can be harnessed for health promotion [61,62,63]. Traditional healthcare settings often stigmatize people who use drugs [64] and therefore many people who use drugs avoid using them [65]. In contrast, HRSOs can provide safe spaces where people go to acquire prevention resources and not experience stigma [66]. Given that the few participants who knew about or used both fentanyl test strips and naloxone obtained this information from HRSOs, and not emergency rooms, healthcare staff, etc., future interventions should strongly consider working with HRSOs.

Most participants described using cold water as an overdose response and did not discuss the use of naloxone when witnessing or personally experiencing an overdose because many did not actually have the medication. Broadly speaking, states with similar naloxone access laws granting direct authority to pharmacists to provide naloxone tend to experience declines in fatal opioid-related overdoses [67]. Although pharmacists in Illinois can dispense naloxone via a state issued standing order (e.g., without an individualized prescription) [50], the rural landscape of participating pharmacies may make it more difficult to access due to large distances between participating pharmacies [68], lack of implementation at the pharmacy level, cost of the medication, or due to fears of stigmatization for getting naloxone by pharmacy staff.

One identified barrier to naloxone use was the widespread notion that naloxone would induce an opioid withdrawal. This is becoming more salient as higher dose formulations of intranasal naloxone (e.g., 4 mg, 8 mg) are becoming common, in part due to the presence of fentanyl in the unregulated drug supply. To address this potential for naloxone-induced opioid withdrawal, more people who use drugs are opting for intermuscular naloxone delivery where the dose can be monitored and titrated. Working with people who use drugs to understand the impacts of naloxone dosing and how they affect naloxone willingness should be considered as states begin adopting higher dose naloxone [69]. In addition, education about the effects of naloxone after administration, with a focus on communication skills to mitigate potential post-reversal conflict between the naloxone administrator and the naloxone recipient, could help [70].

Another barrier to naloxone use, as well as to calling emergency services to aid in instances of overdose, was the fear of a negative police response. Illinois has enacted Good Samaritan Laws (GSL) that offer legal protection to people who call 911 or seek medical attention related to an opiate overdose [71]. While these laws have been associated with reductions in opioid related mortality [72], our data, as well as others [73,74], suggest that participants are either unaware of GSL or that distrust and fear of law enforcement remain a barrier to overdose response, despite GSL.

These findings suggest interventions are needed to further support persons who inject drugs to use naloxone and call 911 when witnessing an overdose. Given the widespread fear of legal repercussions associated with overdose responses, guidance and education are needed to inform persons who inject drugs, police, and the public about policies that legalize the possession of naloxone and to limit arrest and prosecution for carrying naloxone with other injection paraphernalia [75,76]. States and localities should expand criminal legal protections for low-level drug related violations when witnesses call 911 for an overdose.

This study is not without limitations. The study sampled persons who inject drugs who were actively using HRSO services. There are many persons who inject drugs who do not use harm reduction services who are not represented in this study, and who may be placed at greater risk of fatal overdose if they do not have access to harm reduction services such as fentanyl testing strips, naloxone, and the latest harm reduction information. Further, the HRSO we partnered with may differ from other HRSOs in the U.S.; therefore, our data may not be generalizable to persons who inject drugs engaged in harm reduction elsewhere. Interviews were conducted before the COVID-19 pandemic and therefore we cannot describe how COVID-19 may have altered the landscape. However, recent studies have shown that the pandemic has exacerbated overdose risk [77,78,79,80], thus making overdose prevention even more important. Given the semi-structured qualitative nature of the study and the interview guide, we did not systematically collect information about length of drug use, age of first use, criminal legal history, or engagement in drug treatment. Some participants discussed these topics, but they came up organically. Therefore, we are unable to provide detailed information on these topics for all participants. Finally, the sample size was limited to 19 participants who injected drugs and 4 service providers, and therefore may not reflect the experiences and perspectives of all people who inject drugs or service providers residing in this region. However, we do believe we met theoretical saturation in our sample, as new themes were not emerging in interviews [42].

Despite these limitations, this study provides a nuanced understanding of overdose risk and prevention strategies in rural southern Illinois. These findings should inform regional-specific interventions to reduce overdose risk among persons who inject drugs.

## 5. Conclusions

People who inject drugs understood fentanyl to be a potential contaminant in their drug supply, both opioids and stimulants, and they actively engaged in harm reduction techniques to try to prevent overdose. Techniques used to mitigate harms and reduce risks for overdose included: buying from trusted drug sellers, using smaller amounts of drugs, not injecting drugs, and using fentanyl test strips. Responses to overdose included: cold water, having the person overdose move around, and naloxone. Interventions to increase harm reduction education and information about evidence-based ways to prevent and respond to overdoses would benefit this community. In addition, increasing and access to biomedical tools such as fentanyl test strips and community drug checking as well as naloxone to reverse overdoses should be considered. Finally, working with communities who use drugs (including drug sellers) and harm reduction organization to prevent fatal overdose may improve interventions.

## Figures and Tables

**Table 1:** Characteristics of People Who Inject Drugs (n = 19).

	N (%)
Gender	
Male	9 (47.4)
Female	10 (52.6)
Age (mean, std)	36.7 (8.1)
Race/Ethnicity	
White	17 (89.4)
Hispanic or Latinx	0 (0.0)
Black or African American	1 (5.3)
Multiracial	0 (0.0)
Not Specified	0 (0.0)
Native American	1 (5.3)
Sexual Orientation	
Heterosexual or Straight	17 (89.4)
Bisexual	2 (10.6)
Gay or Lesbian	0 (0.0)
Not Specified	0 (0.0)
Education	
Less than High School	3
High School or GED	7
Some College	8
Bachelor’s Degree	1
Graduate Degree	0
Not Specified	0
Currently Inject Drugs	19 (100.0)
Drug Preference	
Preferred opioids	7 (36.8)
Preferring stimulants	12 (63.2)

## Data Availability

Data can be made available upon reasonable request.
